# Análisis cuantitativo de la expresión de genes de resistencia a fluconazol en cepas de *Candida albicans* aisladas al ingreso de adultos mayores a una unidad de cuidados intensivos de Manizales, Colombia

**DOI:** 10.7705/biomedica.4723

**Published:** 2020-03-30

**Authors:** Ana Elisa Rojas, Jorge Enrique Pérez, Johan Sebastián Hernández, Yuliana Zapata

**Affiliations:** 1 Grupo de Investigación en Enfermedades Infecciosas, Universidad Católica de Manizales, Manizales, Colombia Universidad Católica de Manizales Grupo de Investigación en Enfermedades Infecciosas Universidad Católica de Manizales Manizales Colombia; 2 Grupo de Investigación Médica, Universidad de Manizales, Manizales, Colombia Universidad de Manizales Grupo de Investigación Médica Universidad de Manizales Manizales Colombia; 3 Grupo de Resistencia Antibiótica de Manizales, Manizales, Colombia Grupo de Resistencia Antibiótica de Manizales Manizales Colombia

**Keywords:** Candida albicans, fluconazol, farmacorresistencia fúngica, farmacorresistencia fúngica múltiple, Candida albicans, fluconazole, drug resistance, fungal, drug resistance, multiple, fungal

## Abstract

**Introducción.:**

Las infecciones oportunistas asociadas con *Candida albicans* han tenido gran repercusión en la salud pública por la mortalidad que generan en determinados grupos poblacionales. Aunque existen tratamientos farmacológicos disponibles, es evidente el aumento de la resistencia desarrollada por el agente patógeno, por lo que la determinación de los mecanismos de resistencia de las cepas presentes en las áreas hospitalarias es importante, ya que permitiría plantear mejores esquemas de tratamiento.

**Objetivo.:**

Analizar la expresión de los genes *ERG11, CDR1* y *MDR1* en cepas de *C. albicans* aisladas de adultos mayores a su ingreso en la unidad de cuidados intensivos del Hospital Santa Sofía de Manizales, Colombia.

**Materiales y métodos.:**

Se seleccionaron 29 muestras (21 resistentes y 8 sensibles) y se conformaron dos grupos de trabajo, uno de muestras con exposición al fluconazol y el otro sin esta. El ARN extraído se cuantificó mediante reacción en cadena de la polimerasa con transcriptasa inversa en tiempo real (RT-qPCR).

**Resultados.:**

Se encontraron diferencias significativas en la expresión del gen *MDR1* en el grupo de cepas de *C. albicans* resistentes. Dos de las cepas resistentes (104 y 62-2) expuestas al antifúngico presentaron valores muy elevados en la expresión de este gen. La expresión del *ERG11* y del *CDR1* no fue significativa en los grupos estudiados.

**Conclusión.:**

El aumento de sobreexpresión del gen *MDR1* indica que este puede ser el responsable de la resistencia; sin embargo, algunas cepas resistentes no sobreexpresaron los genes analizados, lo que indica que puede haber otros genes involucrados en la resistencia de las cepas estudiadas.

## Abstract (en)

**Introduction:**

Opportunistic infections associated with *Candida albicans* have had a great impact on public health due to the mortality they generate in certain population groups. Although pharmacological treatments are available, the resistance developed by the pathogen has become increasingly evident. For this reason, determining the mechanisms of resistance associated with the strains found in different hospital areas is important since it would help improving treatment plans.

**Objective:**

To analyze the expression of *ERG11, CDR1,* and *MDR1* genes in strains of *C. albicans* isolated from elderly patients at admittance in the intensive care unit of *Hospital Santa Sofía* in Manizales, Colombia.

**Materials and methods:**

A total of 29 samples (21 resistant and 8 sensitive) were selected and distributed in two working groups: with and without exposure to fluconazole. The extracted RNA was quantified by real-time reverse transcription polymerase chain reaction (RT-qPCR).

**Results:**

Significant differences were found in the expression of the *MDR1* gene in the group of resistant *C. albicans* strains. Two of the resistant strains (104 and 62-2) exposed to the antifungal showed very high values in the expression of this gene. The expression of *ERG11* and *CDR1* was not significant among the groups studied.

**Conclusion:**

The increased overexpression of the *MDR1* gene indicates that it may be responsible for the resistance. However, some resistant strains did not overexpress any of the genes analyzed, which indicates that there may be other genes involved in the resistance of the strains under study.

Varias especies de *Candida* son comensales, y colonizan la piel y las superficies mucosas de los seres humanos. Los pacientes gravemente enfermos o inmunocomprometidos son más propensos a desarrollar infecciones superficiales y sistémicas potencialmente mortales ^1^.

En el tratamiento de infecciones por *Candida* spp., los azoles han sido el tratamiento de primera línea. El fluconazol el más usado de este grupo, pues se le considera el primer antifúngico sistémico con toxicidad reducida y con un excelente perfil farmacocinético; además, tiene presentaciones para formulación oral o intravenosa ^2^^,^^3^.

En Colombia, la frecuencia de *Candida* spp. en hemocultivos es del 5 % y es el quinto germen más frecuentemente aislado ^4^. En los estudios realizados en nuestro país entre el 2003 y el 2015, se ha reportado que la frecuencia de resistencia de *C. albicans* al fluconazol varía entre el 1,1 y el 8,6 %, ^5^^-^^9^. Los datos más recientes indican que el porcentaje de resistencia a los azoles sigue siendo bajo y permanece estable. Sin embargo, a nivel regional dicha resistencia se ha estudiado poco y, en Caldas, no se conocen los mecanismos de resistencia responsables de la resistencia de las especies de *Candida.*

El primer mecanismo asociado con la disminución de la sensibilidad de *Candida* spp. a los azoles es la inducción de bombas de expulsión que disminuyen la concentración del fármaco en la célula fúngica ^10^^,^^11^. El primer grupo de estas proteínas de membrana corresponde a las CDR, grupo de transportadoras de tipo ABC *(ATP binding cassette),* que expulsan diferentes compuestos mediante un mecanismo activo dependiente de ATP. En *C. albicans* se han caracterizado dos de estos genes, el *CDR1* y el *CDR2,* los cuales se sobreexpresan en más del 50 % de las cepas resistentes a los medicamentos antifúngicos ^12^.

El gen *MDR1* codifica la proteína de 564 aminoácidos conocida como Mdr1, la cual es un transportador de tipo I de diferentes moléculas, entre ellas, los medicamentos. Esta proteína pertenece a la superfamilia de facilitadores principales *(Major Facilitator Superfamily,* MFS). Se ha reportado que su sobreexpresión contribuye al flujo externo de los azoles usualmente empleados para tratar la infección por *C. albicans*^13^.

Otro mecanismo común de resistencia en las especies de *Candida* es la sobreexpresión del gen que codifica la enzima diana *(ERG11),* lo que incrementa los niveles de la enzima desmetilasa de 14a lanosterol en la membrana del hongo y puede llevar a que los azoles no tengan afinidad por la enzima ^14^^-^^16^.

El objetivo del presente trabajo fue caracterizar por primera vez en la región de Caldas la expresión de algunos de los factores de resistencia al fluconazol más descritos en la literatura científica en una selección de los aislamientos incluidos en el primer muestreo para la evaluación de la resistencia antifúngica de especies de *Candida* en las unidades de cuidados intensivos de este departamento.

## Materiales y métodos

### Selección de muestras y grupos de análisis

En los procedimientos se usaron elementos de las guías *Minimum Information for Publication of Quantitative Real-Time PCR Experiments* (MIQE) para este tipo de experimentos de expresión ^17^. De las cepas de *C. albicans* de pacientes colonizados obtenidas previamente en el "Estudio de la colonización de especies de *Candida* en adultos mayores al ingreso de cuidados intensivos" ^9^, se seleccionaron 29: 21 clasificadas como resistentes al fluconazol (concentración inhibitoria mínima, CIM, de 8 µg/ml o más a las 24 horas) y ocho sensibles (CIM de 2 µg/ml o menos a las 24 horas).

Las cepas seleccionadas se repicaron en agar PDA y se identificaron a nivel de especie, siguiendo los protocolos del laboratorio. Se confirmó su CIM frente al fluconazol, utilizando el método de microdilución para levaduras propuesto por *The Clinical and Laboratory Standards Institute* (CLSI) en su manual M27-A3 ^18^.

### Ensayo de macrodilución con fluconazol y sin fluconazol

Cada cepa fue sometida a una prueba de macrodilución siguiendo el protocolo propuesto por el CLSI y utilizando el medio líquido de cultivo Sabouraud; todos los tubos inoculados se incubaron con agitación constante a 35 °C hasta el momento en el que se produjo la fase logarítmica de crecimiento de las levaduras, cuando se hizo la lectura de cada uno de los tubos para establecer su CIM, comparando el tamaño del sedimento celular de cada concentración de fluconazol con el control positivo.

Se recolectaron las células del tubo en el que se presentó un crecimiento celular mayor del 50 %, se ajustó la concentración celular a un valor entre 2 x 10^8^ y 3 x 10^8^ levaduras, proceso que también se realizó con el tubo correspondiente al control positivo. Cada tubo empleado se centrifugó para obtener el sedimento de levaduras, el cual se recolectó en un tubo libre de ADNasa y ARNasa, y se conservó en solución RNAlater™ (Ambion, ThermoFisher) y se congeló a -80 °C hasta el momento de la extracción del ARN.

### Extracción de ARN

Cada uno de los tubos con el sedimento de levaduras se sometió al proceso de extracción de ARN utilizando el estuche RiboPure RNA™, yeast (Invitrogen), ampliamente usado en la extracción de ARN en *C. albicans,* y siguiendo el protocolo descrito por el fabricante.

### Criterios de calidad del ARN extraído

Se hicieron lecturas de la absorbancia de las muestras a 230, 260 y 280 nm en un espectrofotómetro UVIS Drop UVS99™ (Avans Biotechnology). Aquellos ARN con relaciones de A_260_:A_280_ y A_260_:A_230_ mayores o iguales a 2, se consideraron de alta pureza y fueron posteriormente utilizados en la reacción en cadena de la polimerasa con transcriptasa inversa en tiempo real (RT-qPCR). Para establecer el grado de integridad del ARN obtenido, se hizo una electroforesis en gel de agarosa al 1 % y se consideró que el ARN no estaba degradado cuando aparecieron dos bandas en el gel.

### Estandarización del protocolo de expresión del ARN mensajero medianteRT-qPCR

Para estandarizar y evaluar la eficiencia de los cebadores, se utilizó la cepa de *C. albicans* ATCC 90028 como referencia de cepa sensible y se eligió al azar una cepa del grupo resistente al fluconazol. El protocolo se cumplió en cada una de las cepas elegidas de los grupos de trabajo propuestos.

### Selección y optimización de cebadores para la qPCR

Los cebadores para la reacción del gen de control endógeno de referencia *(ACT1)* y de los genes de resistencia *(ERG11, MDR1, CDR1)* (cuadro 1) fueron los propuestos por Chau, *et al.*^19^.

**Cuadro 1:** Secuencia de cebadores usados en los ensayos de qPCR

Gen		Secuencia del cebador (5’-3’)
*ACT1*	Hacia adelante	TTGGTGATGAAGCCCAATCC
	Hacia atrás	CATATCGTCCCAGTTGGAAACA
*MDR1*	Hacia adelante	TTACCTGAAACTTTTGGCAAAACA
	Hacia atrás	ACTTGTGATTCTGTCGTTACCG
*CDR1*	Hacia adelante	TTTAGCCAGAACTTTCACTCATGATT
	Hacia atrás	TATTTATTTCTTCATGTTCATATGGATTGA
*ERG11*	Hacia adelante	GGTATTGGCTGGTCCTAATGTGA
	Hacia atrás	GCTTGAATCAAATAAGTGAATGGATTAC

Para la prueba de optimización de los cebadores, se utilizó 1 µl del ADN complementario, y las temperaturas ideales de alineamiento y concentración optima de cebadores para cada uno de los genes se evaluaron con el reportero SYBR Green™ (Applied Biosystems), siguiendo los pasos propuestos por Chau, *et al.*^19^. El análisis de la temperatura de disociación *(melting temperature,* Tm) permitió evaluar la especificidad de los cebadores.

### Protocolo de la transcripción inversa del ARN total a ADN complementario

El proceso de transcripción inversa se hizo separadamente de las reacciones de la qPCR, utilizando el estuche SensiFAST cDNA™ (Bioline) y siguiendo sin modificaciones las indicaciones del fabricante. La reacción de transcripción inversa se realizó en el termociclador StepOnePlus Real-Time PCR System™ (Applied Biosystems) con el siguiente protocolo: incubación a 25 °C durante 10 minutos, luego a 50 °C durante 30 minutos y, finalmente, se inactivó la enzima a 85 °C durante 5 minutos.

### Experimento de qPCR

Los análisis de PCR en tiempo real se llevaron a cabo en el equipo StepOnePlus™ (Applied Biosystem) utilizando el estuche Power Up SYBR Green Master Mix™ (Applied Biosystem). Para evaluar la expresión a nivel de la transcripción de los genes *ERG11, CDR1* y *MDR1,* se utilizó como gen de referencia el *ACT1.* La especificidad de los productos de las reacciones de q-PCR se evaluó mediante el análisis de las curvas de disociación. La cepa de *C. albicans* ATCC 90028 se utilizó como referencia.

Cada reacción de cuantificación mediante q-PCR contenía 10 µl de PowerUp SYBR Green Master Mix 2x™, 1,6 µl de cada pareja de cebadores en una concentración final de 0,8 µM (cuadro 1), 2 µl de ADN complementario, y el volumen de 20 µl se completó con agua tratada con DEPC (Thermo Scientific).

El programa de amplificación utilizado consistió en una desnaturalización inicial con dos pasos: a 50 °C durante dos minutos y a 95 °C durante dos minutos, seguidos de 40 ciclos de desnaturalización a 95 °C durante 15 segundos y una extensión a 60 °C durante un minuto. Con el fin de establecer la curva de disociación del ADN complementario e identificar las reacciones de amplificación no específicas, se utilizaron los siguientes parámetros: 95 °C durante 15 segundos, 60 °C durante un minuto y 95 °C durante 15 segundos.

### Análisis de la reacción de qPCR

Se utilizó el método de análisis comparativo ACt propuesto por Livak ^20^ para establecer la diferencia entre el ciclo de cruce del umbral de detección (Ct) de los genes de resistencia *(ERG11, MDR1* y *CDR1)* y el gen de control endógeno de referencia (ACT1); el análisis de los resultados se hizo con el programa StepOnePlus Real-Time PCR™ (Applied Biosystems). Este mismo programa permitió evaluar la eficiencia de cada una de las reacciones. Los genes de cada una de las muestras se analizaron por triplicado.

### Análisis estadístico

En el análisis estadístico de los datos, se usó la prueba no paramétrica de Wilcoxon pareada mediante el programa R. El valor de p<0,05 se consideró como estadísticamente significativo.

## Resultados

### Cuantificación relativa de los genes ERG11, CDR1 y MDR1 mediante q-PCR

La media, la desviación estándar y la mediana obtenidas en la cuantificación relativa (RQ) de cada uno de los genes estudiados en cada uno de los grupos de análisis, se presentan en el cuadro 2. La expresión génica de todos los genes fue muy similar, con excepción del gen *MDR1,* que presentó sobreexpresión en las cepas resistentes, y del gen *ERG11* que la presentó en las cepas sensibles.

**Cuadro 2:** Valores estadísticos de la expresión relativa de los genes *ERG11, MDR1* y *CDR1* en cepas sensibles y resistentes al fluconazol

Variable	n	Media	DE	p (2 colas)*
*ERG11* (R) sin exposición a fluconazol	21	0,42	0,29	0,0724
*ERG11* (R) con exposición a fluconazol	21	0,59	0,63	
*CDR1* (R) sin exposición a fluconazol	21	0,08	0,04	0,1252
*CDR1* (R) con exposición a fluconazol	21	0,1	0,11	
*MDR1* (R) sin exposición a fluconazol	21	1,91	4,8	<0,0001
*MDR1* (R) con exposición a fluconazol	21	64,65	274,23	
*ERG11* (S) sin exposición a fluconazol	8	0,2	0,13	0,0008
*ERG11* (S) con exposición a fluconazol	8	0,48	0,32	
*CDR1* (S) sin exposición a fluconazol	8	0,07	0,07	0,1448
*CDR1* (S) con exposición a fluconazol	8	0,04	0,03	
*MDR1* (S) sin exposición a fluconazol	8	0,42	0,35	0,066
*MDR1* (S) con exposición a fluconazol	8	0,59	0,56	

* Valor de p (2 colas): se obtuvo comparando los valores de cada uno de los genes *(ERG11, CDR1* y *MDR1)* en los grupos de cepas sensibles (S) y resistentes (R), con exposición al fluconazol y sin ella, con el fin de establecer la variación de cada uno de ellos.

**Figura 1:**
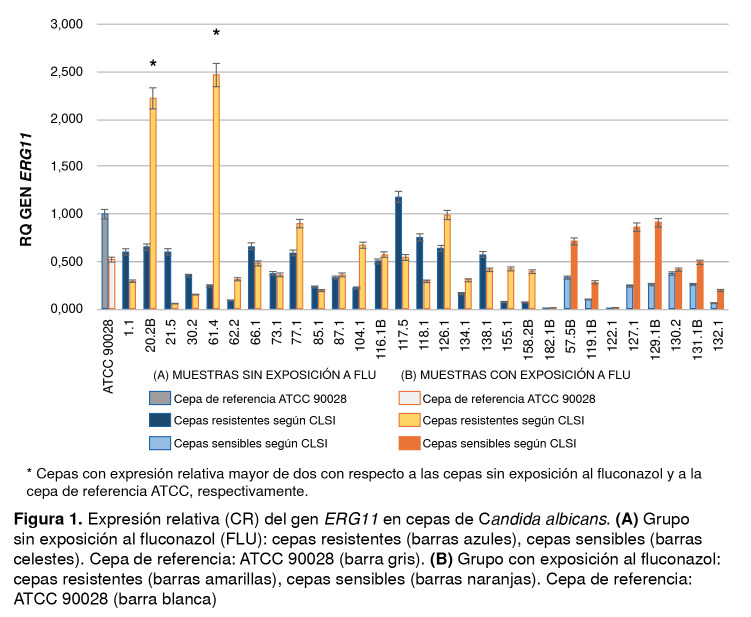
Expresión relativa (CR) del gen *ERG11* en cepas de *Candida albicans.* **(A)** Grupo sin exposición al fluconazol (FLU): cepas resistentes (barras azules), cepas sensibles (barras celestes). Cepa de referencia: ATCC 90028 (barra gris). **(B)** Grupo con exposición al fluconazol: cepas resistentes (barras amarillas), cepas sensibles (barras naranjas). Cepa de referencia: ATCC 90028 (barra blanca)

El promedio de la expresión del gen *ERG11 e*n todos los grupos estudiados tuvo un valor por debajo de la cepa de referencia ATCC; solo dos muestras del grupo de resistentes expuestas al fluconazol (20-2B y 61-4) generaron una expresión relativa mayor de 2, en tanto que nueve de estas cepas presentaron una sobreexpresión menor (62-2, 77-1, 87-1, 104-1, 116-1, 126-1,134-1, 155-1 y 158-2B). Siete de las ocho muestras sensibles también presentaron un aumento en la expresión del gen al exponerlas al fluconazol (57-5B, 119-1B, 127-1, 129-1B, 130-2, 131-1B y 132-1) (figura 1).

En el análisis estadístico, no se observaron diferencias significativas (p=0,96) en la expresión de este gen al comparar las cepas resistentes con las sensibles; sin embargo, al comparar los valores de expresión de las cepas expuestas con las no expuestas, sí se encontró una diferencia significativa en las cepas sensibles mas no en las resistentes (p=0,0008 y p=0,072) (cuadro 2).

Al analizar la expresión del gen *MDR1* en las cepas resistentes bajo exposición al antifúngico, se encontró que nueve de ellas presentaban un aumento en la expresión de este gen y, en tres de estas (30-2, 62-2 y 104-1), con valores relativos por encima de 20 (cuadro 3). Al comparar los valores de este gen en este grupo con fluconazol y sin fluconazol, se encontró una diferencia estadísticamente significativa (p<0,05) (cuadro 2). En el grupo de las cepas sensibles, tres presentaron un aumento en la expresión de este gen, pero solamente una con valores por encima de uno (cuadro 3). No se encontraron diferencias significativas al comparar los resultados de las expuestas con las de las no expuestas (cuadro 2). Al comparar los valores de las cepas sensibles con las resistentes no expuestas al fluconazol, se observó que no había significación estadística (p=0,1071). El mismo comportamiento se encontró al comparar las cepas sensibles y las resistentes expuestas al fluconazol (p=0,33).

**Cuadro 3:** Cuantificación relativa del gen MDR1 en cepas de *Candida albicans,* resistentes y sensibles al fluconazol, con exposición al antifúngico y sin ella

Perfil de sensibilidad	No de muestra	Valor promedio de CR sin exposición a fluconazol	Valor promedio de CR con exposición a fluconazol
	ATCC 90028	1,000	0,750
R	1-1	1,950	1,470
R	20-2B	0,640	0,540
R	21-5	0,370	0,000
R	30-2	0,16	20,88
R	61-4	1,110	3,680
R	62-2	1,125	1260,01
R	66-1	0,590	0,620
R	73-1	1,580	0,460
R	77-1	1,330	1,570
R	85-1	1,420	0,990
R	87-1	0,280	0,730
R	104-1	22,64	61,21
R	116-1B	2,370	1,010
R	117-5	0,300	0,100
R	118-1	0,350	0,090
R	126-1	1,560	1,390
R	134-1	1,370	1,940
R	138-1	0,400	0,230
R	155-1	0,400	0,620
R	158-2B	0,130	0,120
R	182-1B	0,000	0,000
S	57-5B	0,000	0,000
S	119-1B	0,520	1,750
S	122-1	0,000	0,020
S	127-1	0,760	0,740
S	129-1B	0,490	0,660
S	130-2	1,000	0,750
S	131-1B	0,380	0,590
S	132-1	0,230	0,250

CR: cuantificación relativa; R: resistente; S: sensible

**Figura 2:**
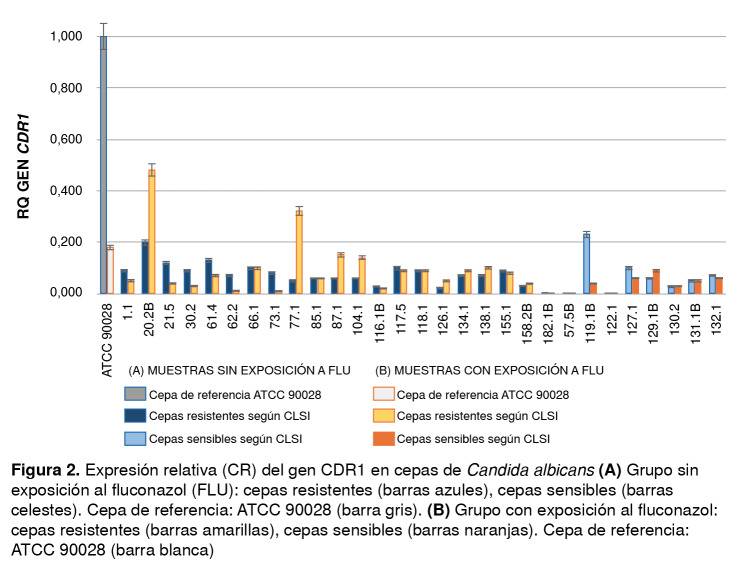
Expresión relativa (CR) del gen CDR1 en cepas de *Candida albicans* **(A)** Grupo sin exposición al fluconazol (FLU): cepas resistentes (barras azules), cepas sensibles (barras celestes). Cepa de referencia: ATCC 90028 (barra gris). **(B)** Grupo con exposición al fluconazol: cepas resistentes (barras amarillas), cepas sensibles (barras naranjas). Cepa de referencia: ATCC 90028 (barra blanca)

En cuanto al gen *CDR1,* se evidenció que seis de las 21 cepas resistentes al fluconazol (20-2B, 77-1, 87-1, 104-1, 134-1 y 138-1) presentaron un aumento en su expresión al compararlas con las no expuestas (figura 2), aunque ninguno de estos valores fue mayor de 1. Al comparar los valores obtenidos en estas cepas con exposición al medicamento y sin ella, no se observó una variación significativa en la expresión del gen (p=0,1252); un comportamiento similar se presentó en el grupo de las cepas sensibles (p=0,1448) (cuadro 2). Al comparar el grupo de cepas resistentes con el de las sensibles expuestas y no expuestas al antimicótico, tampoco se encontró significación estadística (p=0,2699 y p=0,11).

Al analizar los datos obtenidos en las cepas resistentes, se observó que cuatro sobreexpresaron tres genes (77-1, 87-1, 104-1, y 134-1); seis cepas sobreexpresaron dos genes (20-2B, 61-4, 62-2, 126-1, 155-1 y 158-2B), y tres cepas sobreexpresaron un solo gen (30-2, 66-1 y 116-1B).

## Discusión

El objetivo del estudio fue caracterizar los principales mecanismos moleculares de resistencia al fluconazol en cepas clínicas de *C. albicans,* una levadura comensal oportunista responsable del 42 % de las infecciones por levaduras en Colombia ^21^. En los estudios previos se había evaluado la sensibilidad de *Candida* spp. a diferentes antifúngicos; sin embargo, hasta ahora no se había reportado ningún estudio regional que identificara los mecanismos moleculares de resistencia o que estableciera su frecuencia en cepas de *C. albicans* aisladas de instituciones de salud. En este contexto, el presente estudio permite entender de manera preliminar los mecanismos de resistencia al fluconazol que se están generando a nivel local.

La estandarización de la metodología de cuantificación de los genes mediante q-PCR y su análisis en diferentes cepas de *C. albicans,* representan el primer paso en la correcta cuantificación relativa de los genes asociados con la resistencia. Se siguió un protocolo que garantizaba las condiciones necesarias para establecer la especificidad de cada pareja de cebadores y optimizar las reacciones de q-PCR, la concentración de cebadores y las temperaturas de los ciclos de amplificación, con el fin de obtener eficiencias entre el 90 y el 100 %.

Un segundo objetivo consistió en evaluar el grado de expresión de los genes *ERG11, CDR1* y *MDR1* en las cepas resistentes y sensibles al fluconazol, expuestas y no expuestas al antifúngico. En varios estudios se ha reportado que es común encontrar que estos genes se sobreexpresan en presencia de ciertos medicamentos antifúngicos ^14^^,^^22^. Se ha comprobado que este tipo de sobreexpresión es independiente del tipo de antifúngico que se use en las pruebas. En este estudio, se evidenció que en solo cuatro aislamientos resistentes expuestos al fluconazol se sobreexpresaron los tres genes, pero no se descarta que en las cepas estudiadas existan mutaciones de dichos genes que no permitan una expresión en conjunto.

La cuantificación de los transcritos del gen *ERG11* en las cepas resistentes y en las sensibles al fluconazol demostró que, si bien en dos cepas resistentes (20-2B, 61-4) hubo un aumento considerable de la expresión en comparación con las demás, en general, la expresión de *ERG11* no presentó diferencias significativas entre las cepas resistentes, las expuestas y las no expuestas; además, en siete de las ocho cepas sensibles hubo sobreexpresión del gen (figura 1), lo cual sugiere que este no sería el mecanismo responsable de la resistencia en las cepas analizadas. Estos resultados concuerdan con los de algunas publicaciones que mencionan que la sobreexpresión del *ERG11* no estaría claramente asociada con la generación de resistencia de *C. albicans*^19^^,^^23^^-^^25^. Sin embargo, en otras especies intrínsecamente resistentes a los azoles, por ejemplo, C. *krusei,* la sobreexpresión de este gen *sí* se ha asociado con resistencia ^26^.

En los estudios de Hiller, *et al.,* se demostró que la acción del gen *MDR1* es suficiente para conferir resistencia a *C. albicans* contra medicamentos como el fluconazol ^27^. En el presente estudio, se encontró que nueve cepas resistentes expuestas al medicamento presentaron sobreexpresión de este gen y, al comparar los datos con las cepas no expuestas, se encontró significación estadística. En un estudio anterior realizado por nuestro grupo de investigación, la cepa 104-1 presentó la mutación G464S del gen *ERG11,* mutación que en diferentes estudios se ha relacionado con la resistencia al fluconazol ^28^. A pesar de que esta cepa presentó sobreexpresión de los tres genes, dicho valor fue mayor con el *MDR1,* por lo cual los resultados obtenidos indicarían que dicha cepa tendría cuatro factores de resistencia, de los cuales dos serían preponderantes en la generación de este tipo de reacción al fluconazol.

La función del *MDR1* como un gen de resistencia a múltiples fármacos en *C. albicans* se ha establecido en diversos estudios, en los que se evidencia una fuerte correlación entre la sobreexpresión y la resistencia ^14^^,^^23^^,^^29^^-^^33^. Se ha reportado que la sobreexpresión del *MDR1* en cepas resistentes está mediada por mutaciones de los elementos de regulación en trans y, en investigaciones realizadas a nivel de los elementos en cis, también se ha reportado que las mutaciones puntuales en esas regiones contribuyen a la generación de resistencia ^34^.

En los estudios en los que se ha inactivado el gen *MDR1,* se ha producido una pérdida parcial o completa del fenotipo resistente ^35^^,^^36^. Sin embargo, en otros estudios se ha observado que este gen generalmente se sobreexpresa con otros genes que le confieren resistencia a *C. albicans* frente a diferentes fármacos, lo que sugiere que la sola sobreexpresión del *MDR1* no sería suficiente para mediar la resistencia a algunos medicamentos y que esta dependería de alteraciones adicionales ^37^^-^^39^. Esto explicaría parcialmente los resultados obtenidos en este estudio pues, aunque hubo significación estadística en la expresión de este gen en las cepas resistentes, solamente en nueve de las veintiún cepas hubo sobreexpresión del gen, en tanto que en cuatro de los aislamientos se sobreexpresaron los otros genes analizados en este estudio. Por ello, no se puede afirmar que este gen sea el único responsable de la resistencia al fluconazol en las cepas analizadas y son necesarios más estudios que permitan profundizar los diferentes aspectos de la resistencia de estas cepas.

Con respecto a la expresión del gen *CDR1,* a diferencia de investigaciones anteriores ^22^^-^^25^^,^^40^, en este estudio no se observó correlación entre la resistencia al fluconazol y los niveles de expresión del gen. Aunque ocho de los aislamientos clasificados como resistentes y expuestos al antifúngico presentaron sobreexpresión, su valor no sobrepasó el obtenido en la cepa de referencia y, al compararlos con los datos de los aislamientos sensibles y expuestos al fluconazol, no mostraron significación estadística. Este resultado evidencia que la transcripción de este gen no estaría implicada en la resistencia de las cepas analizadas en el presente estudio y es similar al obtenido por Chau, *et al.,* y Cernicka, *et al.*^40^^,^^41^, quienes no encontraron una correlación determinante entre el aumento de la transcripción del gen y la disminución de la sensibilidad en las cepas estudiadas.

Los hallazgos del presente estudio sustentan la necesidad de secuenciar los genes *MDR1* y *CDR1* para determinar las implicaciones de las mutaciones en ausencia o en presencia de resistencia en las cepas encontradas en los hospitales locales, mutaciones que se han descrito en diversos estudios ^42^^,^^43^. Ello permitiría un mejor enfoque y un mayor conocimiento de la resistencia de *C. albicans.*

En el presente estudio, hubo varias limitaciones. La primera se refiere a la procedencia de las cepas, todas ellas de pacientes colonizados, por lo que los resultados obtenidos no se podrían extrapolar a lo que estuviera ocurriendo a nivel clínico; sin embargo, la presencia de levaduras resistentes con niveles de expresión relativa mayores de 2 en diferentes genes, sería un factor de riesgo para la candidiasis de origen endógeno resistente al tratamiento con fluconazol en estos pacientes, lo que podría eventualmente complicar su situación clínica debido a la facilidad con la que estas levaduras sobreexpresan uno o varios de los genes mencionados; además, la presencia de estas cepas en las mucosas es un riesgo adicional de transmisión a otras personas y, por lo tanto, de dispersión de la resistencia. En este contexto, podría decirse *a priori* que uno de los mecanismos de resistencia más frecuentes estaría asociado con el gen *MDR1.*

La ausencia de muestras de pacientes con candidiasis sistémica y resistencia al fluconazol, no permite confirmar la asociación de dicha resistencia con los genes analizados. Aunque el número de muestras resistentes en este estudio fue baja, si se pudo establecer que en cinco de los veintiún aislamientos los factores de resistencia, se expresaron a concentraciones que permiten confirmar la resistencia presentada por estas cepas al antifúngico *in vitro.*

Por último, el presente análisis se limitó al efecto asociado con un solo antifúngico, lo que impidió obtener resultados mucho más contundentes con respecto al perfil de sensibilidad de las cepas analizadas.

A pesar de estas limitaciones, los resultados obtenidos permiten un análisis preliminar del posible papel causal de la exposición al fluconazol en el desarrollo de la resistencia de los aislamientos de *C. albicans,* lo que validaría la recomendación de evitar la prescripción innecesaria e inadecuada del antifúngico y su uso a largo plazo, el cual debe basarse en la confirmación del diagnóstico ^44^.

En resumen, es evidente que más de un mecanismo molecular puede contribuir al fenotipo de resistencia global en muchos aislamientos. Algo similar se ha observado en varios estudios ^22^^,^^23^. La aparición de múltiples mecanismos de resistencia probablemente refleja los largos períodos de exposición al fármaco y el modo de acción estático de los azoles frente a las levaduras. Es necesario, entonces, continuar analizando las cepas resistentes procedentes de personas colonizadas y las de pacientes que no mejoran adecuadamente con el tratamiento antimicótico, con el fin de entender mejor los mecanismos moleculares más frecuentemente asociados con la resistencia a estos medicamentos en la región y, así, establecer protocolos de tamización temprana de las cepas resistentes para garantizar una mayor efectividad del tratamiento y una disminución de costos al usar medicamentos realmente efectivos.
